# Heavy metal toxicity in plants and the potential NO-releasing novel techniques as the impending mitigation alternatives

**DOI:** 10.3389/fpls.2022.1019647

**Published:** 2022-09-23

**Authors:** Anjali Pande, Bong-Gyu Mun, Nusrat Jahan Methela, Waqas Rahim, Da-Sol Lee, Geun-Mo Lee, Jeum Kyu Hong, Adil Hussain, Gary Loake, Byung-Wook Yun

**Affiliations:** ^1^ Laboratory of Plant Molecular Pathology and Functional Genomics, Department of Plant Biosciences, School of Applied Biosciences, College of Agriculture & Life Science, Kyungpook National University, Daegu, South Korea; ^2^ Laboratory of Horticultural Crop Protection, Department of Horticultural Science, Gyeongsang National University, Jinju, South Korea; ^3^ Department of Entomology, Abdul Wali Khan University, Mardan, Pakistan; ^4^ Institute of Molecular Plant Sciences, The University of Edinburgh, Edinburgh, United Kingdom

**Keywords:** heavy metal toxicity, nitric oxide, NO donors, NO-release, nanoparticles, encapsulation, agriculture

## Abstract

Environmental pollutants like heavy metals are toxic, persistent, and bioaccumulative in nature. Contamination of agricultural fields with heavy metals not only hampers the quality and yield of crops but also poses a serious threat to human health by entering the food chain. Plants generally cope with heavy metal stress by regulating their redox machinery. In this context, nitric oxide (NO) plays a potent role in combating heavy metal toxicity in plants. Studies have shown that the exogenous application of NO donors protects plants against the deleterious effects of heavy metals by enhancing their antioxidative defense system. Most of the studies have used sodium nitroprusside (SNP) as a NO donor for combating heavy metal stress despite the associated concerns related to cyanide release. Recently, NO-releasing nanoparticles have been tested for their efficacy in a few plants and other biomedical research applications suggesting their use as an alternative to chemical NO donors with the advantage of safe, slow and prolonged release of NO. This suggests that they may also serve as potential candidates in mitigating heavy metal stress in plants. Therefore, this review presents the role of NO, the application of chemical NO donors, potential advantages of NO-releasing nanoparticles, and other NO-release strategies in biomedical research that may be useful in mitigating heavy metal stress in plants.

## Graphical Abstract

Heavy metal toxicity reduces plant growth and productivity. Since traditionally used NO donors do not provide a sustained NO-release, therefore heavy metal toxicity can be mitigated by novel NO-delivery techniques.

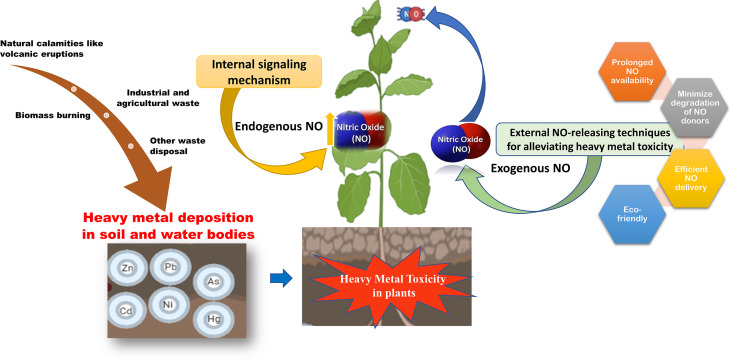

## Introduction

Industrialization and the increasing human population have led to the exploitation of natural resources for anthropogenic activities leading to ecological imbalance. Heavy metal contamination in soil and water is one of the major examples of such human-centric activities that pose a serious threat to the environment (Chen et al., 2022b). However, natural phenomenon like volcanic eruptions and weathering of rocks also contributes to the contamination of soil and water bodies with heavy metals. Contamination of heavy metals in agricultural soil leads to decreased growth and productivity of crops and their bioaccumulation in crops poses serious health hazards as they enter the food chain (Emurotu and Onianwa, 2017). Due to their persistent, bioaccumulative, and toxic nature, these are known as major environmental pollutants (Tchounwou et al., 2012).

Recently, agricultural lands contaminated with heavy metals have gained much attention because of their detrimental effect on the agro-ecosystem. Any adverse effect on the agro-ecosystem directly affects various active and dynamic physical, chemical, and biological activities involved in plant growth and productivity. A principal consequence of heavy metal toxicity in plants is the overproduction of reactive oxygen species (ROS) leading to oxidative stress (Romero-Puertas et al., 2019). However, certain heavy metals like Cadmium (Cd) may not induce ROS production in plants but act as pro-oxidants and suppress the availability of antioxidants (Singh and Shah, 2015; Loix et al., 2017). Thus, heavy metals disturb the equilibrium between the production and scavenging of ROS resulting in oxidative stress (Demecsová and Tamás, 2019). While heavy metals induce the production of ROS, nitric oxide plays an important role in stimulating the antioxidant signaling response in plants, thus alleviating the toxic effects of ROS (Terrón-Camero et al., 2019; Sharma et al., 2020).

Nitric oxide (NO) is a versatile and key molecule known for its role in enhancing plant tolerance to abiotic stresses like drought, salinity, heavy metals, and extreme temperatures (Simontacchi et al., 2015; Begara-Morales et al., 2019; Nabi et al., 2019). Additionally, it plays an important role in various growth and developmental processes in plants such as germination, root development, photomorphogenesis (Corpas and Palma, 2020). Nitric oxide not only activates the antioxidative machinery but also activates the synthesis of phytochelatins which helps plants to cope with the deleterious effects of ROS (Groß et al., 2013). It is also documented that both endogenous and exogenous NO contribute to stress tolerance in plants (Wei et al., 2020). Moreover, the exogenous application of NO (in the form of NO donor) is highly dose-dependent and varies from plant to plant. A detailed account on the dose-dependent application of different NO donors in various plants has been reviewed by Terrón-Camero et al. (2019).

In the last decade, several studies described the use of different NO donors to understand the effects and the mechanism of NO under heavy metal stress in plants (Gill et al., 2013; Singh and Shah, 2014; Imran et al., 2016; Hashem et al., 2018; Li et al., 2019; Piacentini et al., 2020b; Ahmad et al., 2021b). These studies support the notion that the exogenous application of NO donors counterbalances the toxic and detrimental effects of heavy metals on overall plant physiology. Despite the vast application of exogenous chemical NO donors in plants, these are generally unstable and prone to decomposition by high light or temperatures leading to rapid and uncontrolled release of NO which reduces their efficacy (Seabra and Durán, 2010; Seabra et al., 2015). Furthermore, all the NO donors have different molecular weights, different rates of absorption by the plants, and different rates of NO release *in planta*. In addition, some of these NO donors are known to release toxic byproducts along with NO. For example, Sodium Nitroprusside (SNP) is known to release cyanide once absorbed by the plants (Norris and Hume, 1987). Therefore, efforts have been made in developing biomaterials that can release NO in a controlled, efficient, and bio-safe manner. In this context, nanotechnology offers major advantages like easy and efficient encapsulation for better storage and controlled release of such chemicals to the targeted sites.

Recently, the application of nano-NO donors as potential alternatives to chemical NO donors has gained much attention. Studies have reported that the application of nanomaterials enhances the level of endogenous NO, promotes growth, and mitigates environmental stress in plants (Oliveira et al., 2016). Evaluation of the potential of nanoparticles as NO donors have recently begun for agricultural and biomedical purposes. This review aims at discussing the potential advantages of NO-releasing nanomaterials in plants and their usefulness in mitigating heavy metal stress in plants. The review also offers thoughtful insights on the prospects of applying other NO delivery platforms that are so far used in biomedical research but may be useful in plant science as well.

## Heavy metal toxicity and the role of nitric oxide in mitigating heavy metal stress in plants

Heavy metals are serious environmental pollutants due to their acute and chronic toxic effects and widespread occurrence. The most hazardous heavy metals and metalloids in the environment include chromium (Cr), Nickel (Ni), Copper (Cu), Zinc (Zn), cadmium (Cd), Lead (Pb), Mercury (Hg), and Arsenic (As). The toxicity caused by these heavy metals on living organisms depends on the dose and duration of exposure (Chen et al., 2022a). However, certain heavy metals like cd, Pb, and Hg may be toxic even at very low concentrations. Heavy metal toxicity in plants leads to several physiological and morphological changes, responsible for the decline in growth (Chen et al., 2022a). For instance, plants exposed to cadmium showed reduced water and nutrient uptake and a decline in the rate of photosynthesis along with other morphological symptoms like chlorosis, inhibition of growth and browning of root tips that ultimately lead to cell death (Wojcik and Tukiendorf, 2004; Mohanpuria et al., 2007). It has been reported that heavy metals can lead to oxidative deterioration of biological molecules causing DNA fragmentation, lipid peroxidation, and protein oxidation. They can alter the content of antioxidants and may change the antioxidative enzyme activity (Sharma and Dietz, 2009).

**Figure 1:**
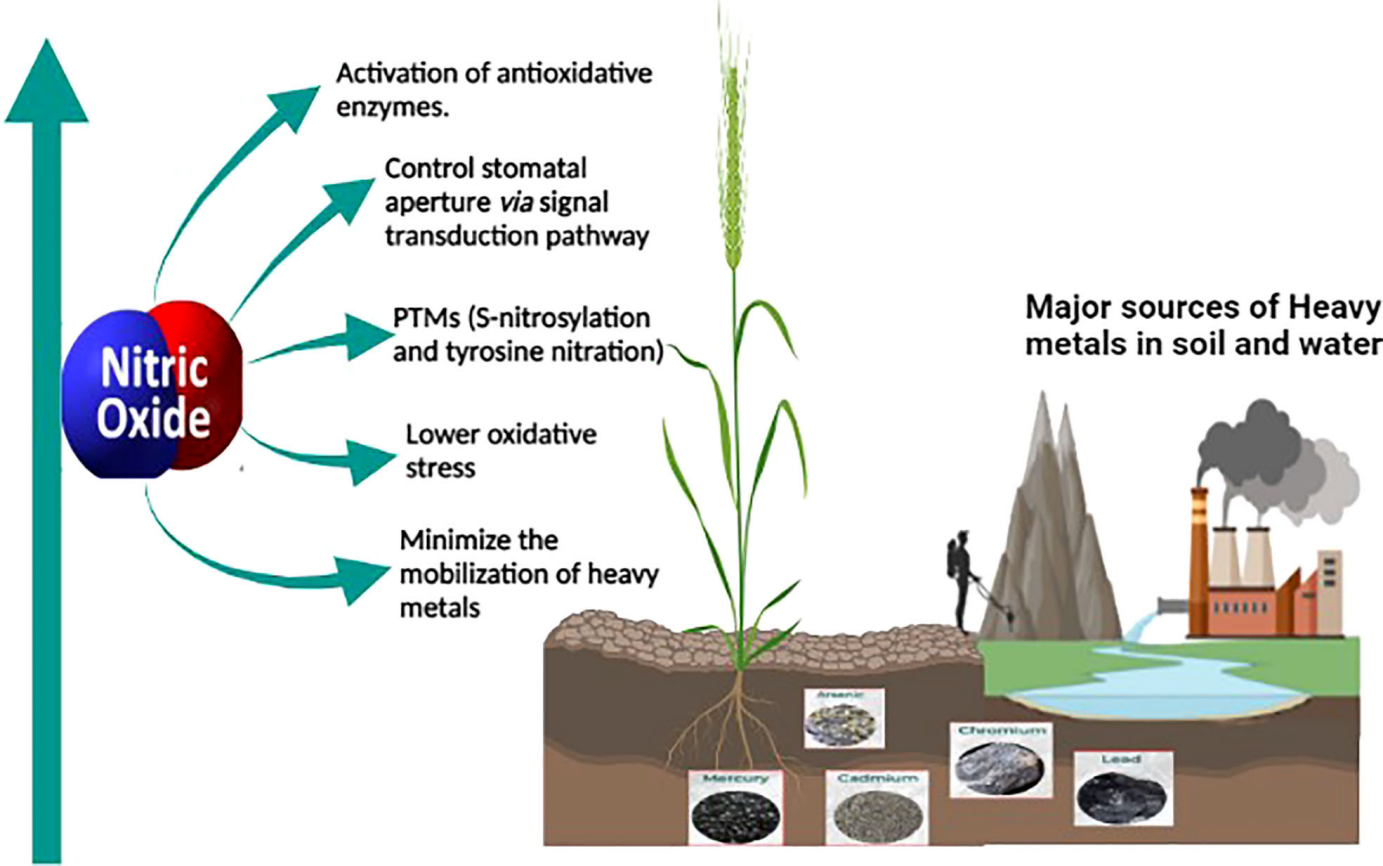
Sources of heavy metal contamination in agricultural land and the role of NO in mitigating heavy metal toxicity in plants. Anthropogenic activities such as industrialization and mining leads to heavy metal contamination in agricultural soil. The oxidative stress caused by the heavy metal toxicity is alleviated by endogenous or exogenously supplied nitric oxide which alleviates it. Nitric oxide is a versatile signaling molecule activating the antioxidative enzymes, controlling stomatal aperture or modifying important proteins through post-translational modification, minimizing mobilization of heavy metals through enhancing the phytochelatins, and thus reducing the toxicity caused by heavy metals.

The role of nitric oxide in mitigating the toxicity induced by heavy metals is well known and thoroughly studied. Nitric oxide (NO) is a key molecule involved in several physiological and biochemical processes in plants. NO is involved in root hair development (Lombardo et al., 2006), enabling plant-microbe interaction during nitrogen fixation (Pande et al., 2021), regulating a balance between auxin and reactive oxygen intermediates (Yu et al., 2014), and for maintaining iron homeostasis (Graziano and Lamattina, 2007). NO also plays a vital role in enhancing the immune response (Tada et al., 2008; Wang et al., 2009) and hypersensitive cell death response (Romero-Puertas et al., 2007; Yun et al., 2011). As a signaling molecule, NO plays a protective role in alleviating abiotic stress conditions (Zhang et al., 2007; Cantrel et al., 2011) including mitigation of heavy metal toxicity in plants (Singh et al., 2016; Nabi et al., 2019; Wei et al., 2020) as shown in **Figure 1**. Most of the studies on heavy metal toxicity in plants indicate that NO reduces the ROS levels by enhancing the levels of antioxidative enzymes (Wang et al., 2010b; Nabi et al., 2019; Terrón-Camero et al., 2019). During heavy metal stress, NO regulates the excessive production of ROS by forming less stable peroxynitrite from the superoxide radical (O_2_^.-^) (Groß et al., 2013). Moreover, NO also regulates the antioxidant enzyme activity in the cell to control the ROS levels during heavy metal stress (Begara-Morales et al., 2019; Khator et al., 2021). Accumulation of NO also leads to the reduction of heavy metal uptake by metal transporters in the roots (Zhao et al., 2013; Singh et al., 2016).

Other studies on heavy metal toxicity suggested the role of nitric oxide in controlling the stomatal aperture (Nabi et al., 2019), in modifying proteins through S-nitrosylation or tyrosine nitration (Saxena and Shekhawat, 2013), and also in minimizing the mobility of heavy metals by enhancing the expression of phytochelatins in plants and thus reducing the heavy metal toxicity in plants (Groß et al., 2013).

Nitric oxide interacts with different biomolecules like phytohormones in response to heavy metal stress in plants. Nitric oxide regulates phytohormonal levels under heavy metal stress conditions. NO is suggested to reduce AsIII toxicity by regulating Jasmonic acid biosynthesis (Singh et al., 2017). It also increases the levels of indole acetic acid (IAA), cytokinins and gibberellic acid while decreasing the levels of ABA in order to lower lead (Pb) uptake and transport (Sadeghipour, 2017).

The interaction between NO and phytohormones is mainly influenced by NO-mediated post-translational modifications (PTMs) under basal as well as induced conditions (Terrile et al., 2012). Protein S-nitrosylation is the most prominent and widely studied PTM among others. It is the selective but reversible redox-based covalent addition of a NO moiety to the sulfhydryl group of cysteine (Cys) molecule(s) on a target protein to form S-nitrosothiols. Our group has recently reviewed a detailed account of phytohormonal regulation through S-nitrosylation under stress (Pande et al., 2022). However, in case of heavy metal stress this is still a potential and important line of inquiry in future.

## NO donors used for alleviating heavy metal toxicity in plants

Exogenous application of NO is most commonly done by supplementing NO donors. Direct application of exogenous nitric oxide to plants is difficult due to its gaseous nature and requires specific equipment (Rodríguez-Ruiz et al., 2017). Moreover, a short half-life (<6 s) of NO makes it difficult to be supplied constantly at the tissue level (Seabra et al., 2015). Therefore, NO is mainly delivered through donor molecules (Wang et al., 2005; Barraud et al., 2009). The commonly used NO donors include SNP, diethylenetriamine NONOate (DETA NONOate), S-nitroso N-acetyl-DL-penicillamine (SNAP), diethylenetriamine/nitric oxide (DETANO), S-nitrosothiols (RSNO), S-nitrosocysteine (CysNO) and S-nitrosoglutathione (GSNO). Recently, a study reported the synthesis and application of N-nitrosomelatonin (NOMela) as a more efficient NO donor than GSNO in Arabidopsis seedlings (Singh et al., 2021a). However, specifically for heavy metal stress tolerance the most commonly used NO donor is SNP (Bothof et al., 2020), treated alone or in combination with other stress ameliorating agents as shown in **Table 1**.

**Table 1:** Studies using NO donors for mitigating heavy metal stress in plants.

NO donor	Plants	Outcome (stress alleviation)	Reference
SNP	*Glycine max*	Mitigation of mercury (Hg) stress.	(Ahmad et al., 2021b)
	*Brassica juncea*	Detoxification of Cd stress.	(Khator et al., 2021)
	*Musa acuminata*	Tolerance against osmotic stress.	(Amnan et al., 2021)
	*Isatis cappadocica*	Improved tolerance to As stress.	(Souri and Karimi, 2021)
	*Vicia faba*	Improved tolerance to As stress.	(Ahmad et al., 2020)
	*Arachis hypogaea*	Inhibition of programmed cell death by aluminum (Al)	(He et al., 2019)
	*Oryza sativa*	Modulation of As toxicity.	(Praveen and Gupta, 2018)
	*Oryza sativa*	Improvement in Ni tolerance.	(Rizwan et al., 2018)
	*Solanum lycopersicum*	Growth promotion under Cd stress.	(Ahmad et al., 2018)
	*Spirodela intermedia*	Alleviation of As stress.	(Da-Silva et al., 2018)
	*Triticum aestivum*	Amelioration of Pb toxicity.	(Kaur et al., 2015)
	*Lolium perenne*	Promotes growth under Pb toxicity.	(Bai et al., 2015)
	*Pogonatherum crinitum*	Controlled Pb uptake.	(Yu et al., 2012)
	*Triticum aestivum*	Mitigation of oxidative stress by enhancing the antioxidative defense response.	(Hasanuzzaman and Fujita, 2013)
	*Arabidopsis thaliana*	Prevention of Lead toxicity in seedlings but no effect on the accumulation	(Phang et al., 2011)
	*Cucumis sativus*	Alleviation of the adverse effects caused by Cd.	(Yu et al., 2013)
	*Lupinus perennis* L.	Mitigation of inhibitory effect of Ni.	(Hassanein et al., 2020)
	*Brassica napus*	Ameliorating Pb toxicity.	(Hamidi et al., 2020)
	*Nasturtium officinale*	Reduction in the adverse effects caused by As.	(Namdjoyan and Kermanian, 2013)
	*Lactuca sativa* var. *capitata*	Reduction in the adverse effects of Co.	(Samet, 2020)
	*Lupinus luteus*	Stimulation of germination and mitigation of inhibitory effects of Cd and Pb stress.	(Kopyra and Gwóźdź, 2003)
	*Triticum aestivum*	Enhancement of root growth under Ni stress.	(Wang et al., 2010b)
	*Capsicum annum*	Reduction in oxidative stress induced by Cd and Pb (applied alone or in combination).	(Kaya et al., 2019)
	*Cicer arietinum*	Reduction in accumulation, toxicity, and oxidative stress induced by Cd.	(Kumari et al., 2010)
	*Typha angustifolia*	Mitigation of Cd stress.	(Zhao et al., 2016)
	*Oryza sativa*	Decreased accumulation of Cd in roots.	(Xiong et al., 2009)
	*Helianthus annuus*	Protection of leaves against Cd-induced oxidative stress.	(Laspina et al., 2005)
	*Lolium perenne*	Mitigation of oxidative stress induced by Cd.	(Chen et al., 2018)
	*Cassia tora* L.	Significant reduction in Al-induced oxidative stress.	(Wang and Yang, 2005)
	*Phaseolus Vulgaris*	Tolerance to Al.	(Wang et al., 2010a)
	*Oryza sativa*	Reduced Cu toxicity and Cu-induced NH_4_^+^ accumulation and Cu toxicity.	(Yu et al., 2005)
	*Solanum lycopersicum*	Alleviation of Cu toxic effects.	(Cui et al., 2010)
	*Triticum aestivum* and *Phaseolus vulgaris*	Maintenance of Zn homeostasis.	(Abdel-Kader, 2007)
	*Hibiscus moscheutos*	Alleviation of inhibitory effects of Al on root elongation.	(Tian et al., 2007)
	*Triticum aestivum*	Alleviation of Cd-induced toxicity and alterations in biochemical factors in roots.	(Singh et al., 2008)
SNP + H_2_O_2_	*Glycine max*	amelioration of As toxicity.	(Singh et al., 2021b)
SNP+ Si	*Brassica juncea*	Mitigation of As stress.	(Ahmad et al., 2021a)
SNP+ Salicylic acid	*Eleusine coracana*	Protection from Ni stress.	(Kotapati et al., 2017)
SNP+ GSH	*Oryza sativa*	Mitigation of the adverse effects of Cu.	(Mostofa et al., 2015)
SNP+ GSH	*Oryza sativa*	Decrease in oxidative stress induced by Cu by enhancing the antioxidative levels.	(Mostofa et al., 2014)
SNP+Auxin	*Oryza sativa*	Mitigation of the adverse effect of Cd stress.	(Piacentini et al., 2020a)
SNP+SA	*Carthamus tinctorius*	Decrease in adverse effects of Zn.	(Namdjoyan et al., 2018)
SNP+TiO_2_ nanoparticles	*Triticum aestivum*	Alleviation of the adverse effects caused by Cd stress.	(Faraji et al., 2018)
SNP+Melatonin	*Catharanthus roseus*	Mitigation of Cd stress.	(Nabaei and Amooaghaie, 2019)
SNP+SA	*Zea mays*	Reduction in negative effects caused by Se.	(Naseem et al., 2020)
SNP ASC + NaNO_2_ N-*tert*-butyl-α-phenylnitrone, 3-morpholinosydonimine (all are NO donors)	*Oryza sativa*	Reduction in CdCl_2_ induced toxicity by reducing oxidative stress	(Hsu and Kao, 2004)

However, due to the relatively unstable nature and susceptibility to decomposition by heat or light, the release of NO is uncontrolled, resulting in unpredictable and random signaling and other physiological effects (Seabra et al., 2015). This problem may be overcome by encapsulating the NO donor molecules of slow and consistent release. Therefore, NO-releasing nanoparticles may be considered as potential alternatives to unstable chemical NO donors.

## Nanoparticles used for alleviating heavy metal toxicity in plants

Nanotechnological interventions in the field of agriculture have paved a way for attaining the long-term goal of sustainable agriculture by improving plant health and productivity under varying environmental conditions (Pande and Arora, 2019). The application and use of nanomaterials not only enhance plant growth and productivity but also help in mitigating biotic and abiotic stress in plants (Arora et al., 2012; Nayan et al., 2016; Bhatt et al., 2020; Zhou et al., 2021). Nanoparticles offer various advantages as compared to their macro counterparts, these include higher surface activity (more surface area available for reaction), enhanced catalytic efficiency, and unique optical and magnetic properties (Wang et al., 2019). Such unique properties add specialized functions to the nanoparticles making them effective in repairing the damage by soil remediation (Liu et al., 2021). The metal adsorption property of magnetite nanoparticles has been found to lower the accumulation of Cd and Na in rice plants (Sebastian et al., 2019). Nanoparticles also influence the formation of apoplastic barriers which suppresses the accumulation of heavy metals in the soil (Rossi et al., 2017). Nanoparticles are useful in mitigating heavy metals in various ways. For instance, they prevent the translocation of heavy metals by forming complexes with them which leads to their immobilization at inactive sites like vacuoles (Wang et al., 2021). These complexes also get adsorbed on the cell surfaces, restricting their movement and biological activity (Cui et al., 2017; Wang et al., 2021). Activation of enzymatic (superoxide dismutase (SOD), catalase (Cantrel et al., 2011), ascorbate peroxidase (APX), glutathione reductase (GR), glutathione peroxidase (GPX), and peroxidase (POD), and non-enzymatic (such as vitamin C, vitamin E, and polyphenols) anti-oxidative defense system is another strategy to cope with the toxicity caused by heavy metals (Zhou et al., 2021). However, a more effective strategy would be to combine the properties of nanoparticles with NO donors as this will have a more profound effect in combating heavy metal stress. In this context, the characteristics properties of nanoparticles like high permeability, film-forming ability, prolonged contact with the active ingredient, and high diffusion would add to the characteristic properties of NO donors for double protection against heavy metal stress.

## Nitric-oxide releasing nanoparticles as potential alternatives to chemical NO donors in alleviating heavy metal toxicity in plants

The limitations associated with NO donors have led to the development of new biomaterials for the controlled and prolonged release of NO into biological systems including plants (Kim et al., 2014; Seabra et al., 2014). Evaluation of NO-releasing nanoparticles has recently begun as an alternative to chemical NO donors for various biomedical and agricultural purposes (Lopes-Oliveira et al., 2019; Ma et al., 2019; Liang et al., 2020; Pieretti and Seabra, 2020; Pelegrino et al., 2020; Pieroni, 2020; Ahmad et al., 2021a). Some of the NO-releasing nanoparticles used in biomedical research have been listed in **Table 2** with their various applications in different plants.

**Table 2:** Recent examples of the advances in the applications of NO-releasing nanoparticles in agriculture and biomedical research.

S.NO.	NO-releasing Nanoparticle	Applied on	Outcome	Reference
1	Alginate/Chitosan (ratio 0.75) encapsulated with GSH	*Zea mays* *Glycine sp*	Sustained and controlled release of NO over several hours. Potentially useful as controlled release systems.	(Pereira et al., 2015)
2	Chitosan nanoparticle encapsulated with S-nitroso-mercaptosuccinic acid	*Zea mays*	Alleviation of salt stress	(Oliveira et al., 2016)
3	GSNO-loaded mineralized CaCO_3_ nanoparticles	Human breast cancer cells, MCF-7	Improvement in therapeutic activity of doxorubicin.	(Lee et al., 2016)
4	Chitosan nanoparticle encapsulated with S-nitroso-mercaptosuccinic acid	*Heliocarpus popayanensis* *Cariniana estrellensis*	Improvement of seedling acclimation and protection of NO donor from thermal and photochemical degradation.	(Lopes-Oliveira et al., 2019)
5	Tetramethoxysilane derived hydrogel-based NO-releasing nanoparticles	Male, Balb/c mice	Reduction in the inflammatory response.	(Williams et al., 2020)
6	NO-releasing S-Nitrosoglutathione-Conjugated Poly (Lactic-Co-Glycolic Acid) Nanoparticles	Mice	Treatment of MRSA (methicillin-resistant staphylococcus aureus) infected cutaneous wounds.	(Lee et al., 2020)
7	Copper-based metal-organic framework as a controlled NO-releasing vehicle	Mice	Therapy for diabetic wounds	(Zhang et al., 2020)
8	Superparamagnetic iron oxide nanoparticles (SPIONS) based NO-releasing nanoparticles	Rat L2 epithelial cells	Reduction in the inflammatory response.	(Shurbaji et al., 2021)
9	NO-releasing chitosan nanoparticles	BALB/c mice	Treatment of cutaneous Leishmaniasis caused by *Leishmania amazonensis*	(Cabral et al., 2021)

**Figure 2:**
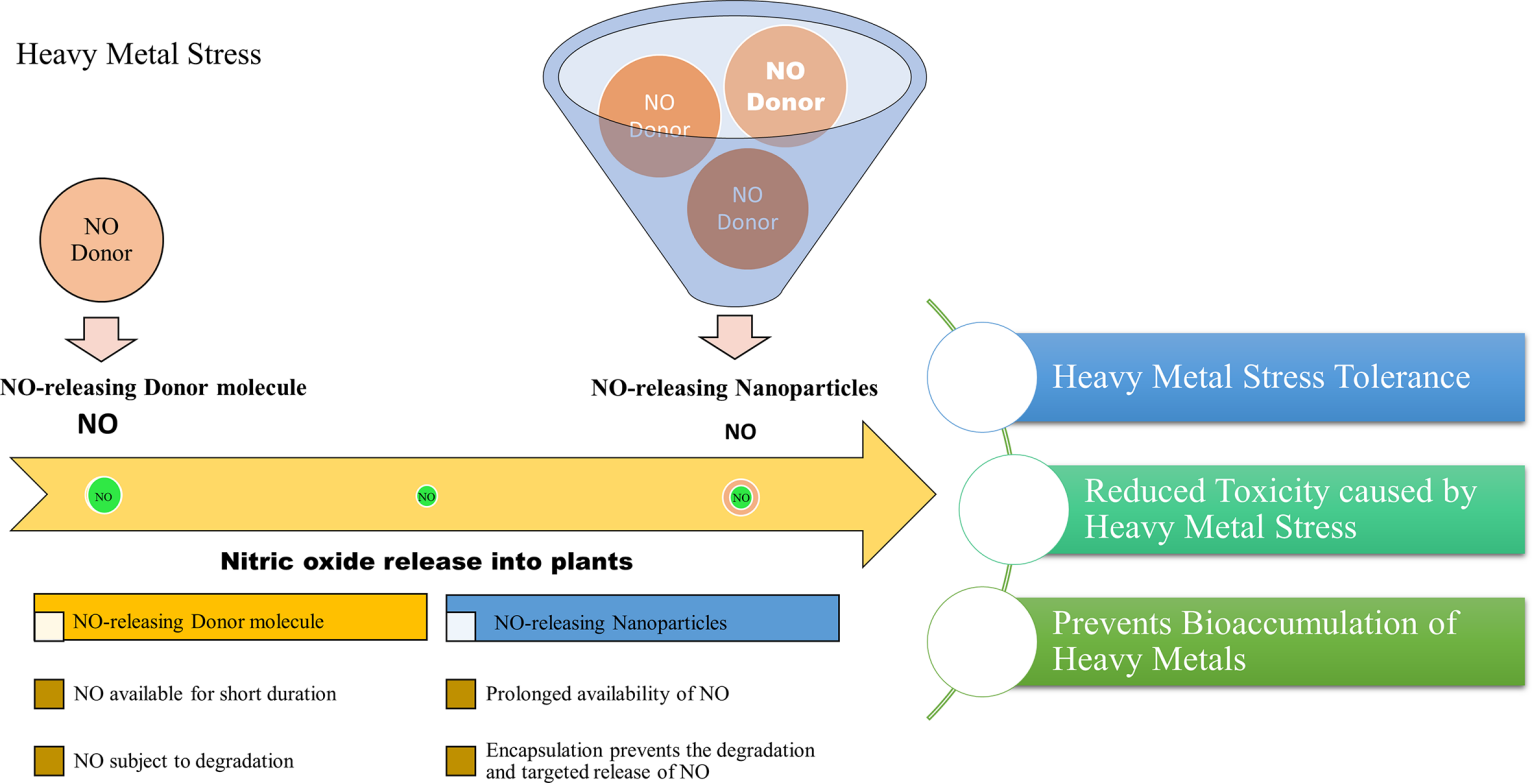
Projected advantages of NO-releasing nanoparticles in alleviating heavy metal toxicity in plants.

A recent study reported the effect of free and nanoencapsulated nitric oxide donor, S-nitroso-mercaptosuccinic acid on neotropical tree seedlings, under field conditions. In this study, the donor molecule was coated with chitosan nanoparticles which protected the molecule from thermal and photochemical degradation. Their study suggested that depending on the tree species, seedling acclimation in the nursery was improved using these nanoencapsulated NO donors (Lopes-Oliveira et al., 2019). Besides, delivering NO is considered a promising approach in biomedical research and applications. The biomedical applications of NO-releasing nanoparticles suggest their importance and also provides insights into their adequacy in plant system as well. Therefore, based on the scientific knowledge we report that the application of NO-releasing nanoparticles is a useful approach to alleviate the detrimental effects of heavy metal stress on plants. The combined effects of nanoparticles and the stress mitigating properties of NO may provide an advantageous approach for combating heavy metal stress in plants. **Figure 2**, demonstrates the advantages of NO-releasing nanoparticles over chemical NO donors in alleviating heavy metal toxicity in plants.

Recent studies have synthesized NO-releasing nanoparticles for studying their effect in plants. NO-releasing nanoparticles are formulated by the addition of NO donor molecules in chitosan nanoparticles which encapsulates the NO donor for slow and prolonged release (Oliveira et al., 2016; Pelegrino et al., 2017a; Pelegrino et al., 2017b; Cabral et al., 2019). These have also been tested for their efficacy in mitigating environmental stress in different plants (Oliveira et al., 2016; Lopes-Oliveira et al., 2019).

## Other NO-releasing techniques used in biomedical research as alternate potential strategies for efficient NO-release in plants

NO is an important signaling molecule that has a significant role in biomedical research. In mammalian tissues, the kinetics and exposure time of NO are key determinants in its biological applications. However, its therapeutic applications are limited due to its extremely short half-life, aimless diffusion into the vasculature, and limited accumulation in the target tissues (Wang et al., 2020).

Traditionally, NO donors were used for NO-release in biomedical sciences which included various types of nitrates, N-diazeniumdiolates, Nitrosothiols, Furoxans, Metal nitrosyl compounds, and Nitrobenzenes (Yang et al., 2021). Nanotechnology has been recently employed for the delivery of NO. In such cases, the nanomaterial is usually degraded once absorbed into the system thereby releasing NO gas through NO donor. Though several different nanotech-based NO delivery platforms have been developed, some interesting studies carry significant potential for application in plant sciences. For example, Lee et al. (2016) described the pH-sensitive release of NO by CaCO_3_ mineralized nanoparticles. This is specifically important in plant sciences as plant growth under basal conditions and soil-related stress conditions such as heavy metal stress, and salinity is significantly related to the pH of the soil. Therefore, such nanoparticle carriers can be engineered to release NO or NO donors at a specific soil pH or under a range of pH conditions. Jia et al. (2018) developed a redox-active nanosilicon-NO donor system that released NO only in response to over-accumulated GSH in tumor tissues. The same concept can be employed in plant sciences by developing nanomaterial-NO donor systems that release NO only in response to over-accumulated chemicals such as salts, heavy metal ions, phytohormones, secondary metabolites or other chemicals/ions in plant tissues. In addition, biomedical researchers developed nanomaterial-NO donors triggered by external cues such as light, heat, X-rays and ultrasound (Fan et al., 2015; Guo et al., 2017; Jin et al., 2017; Hotta et al., 2020; Zhou et al., 2020). Although their clinical applications are limited in mammals, we believe that these may prove to be highly useful in plant sciences. Sunlight is mandatory for photosynthesis. However, intense light for a longer duration and/or certain wavelengths of light are harmful to plant growth. Similarly, intense heat also limits plant growth and development. Nanomaterial-NO delivery systems that release NO under specific light and temperature conditions can be designed for target NO delivery. An alternate biomedical study also suggests the use of nanostructured CuO/SiO_2_ catalysts for releasing NO by the catalytic decomposition of NO-releasing metabolites like GSNO (Kulyk et al., 2020). However, these nanoparticles are suggested to be useful in medical applications and their possibilities and applications need to be explored for agriculture purposes.

Besides, NO donor-conjugated chemical drugs were also designed with more sophisticated NO linkage, release position, selectivity, and amount of NO release. Chen et al. (2008) designed and synthesized multiple NO-releasing derivatives of oleanolic acid (NO-OA) with anti-hepatocellular carcinoma activity. To our knowledge, such types of conjugated NO-donors have not been tested in plants. Moreover, NO release strategies are mostly limited to a few NO donors only (as mentioned in the previous sections) therefore these can be utilized and tested in crop research as well.

The development of targeted prodrugs for gases like NO has been a special challenge in biomedical sciences that carries great prospects. Prodrugs are medications that, after administration, are metabolized and converted into a pharmacologically active drug within the body. Specific enzymes can activate NO prodrugs and release NO gas at specific sites, greatly reducing the side effects. Such types of targeted NO prodrugs developed so far are activated by glycosidases (Wu et al., 2001; Cai et al., 2004), cytochrome enzymes (Saavedra et al., 1997), oxidoreductases (Sharma et al., 2013a), esterases (Saavedra et al., 2000) and reductase enzymes (Sharma et al., 2013b) that trigger the release of NO. Interestingly, plants express a plethora of all these different enzymes in various types of tissues offering the possibility of using NO prodrugs in plant sciences.

**Figure 3:**
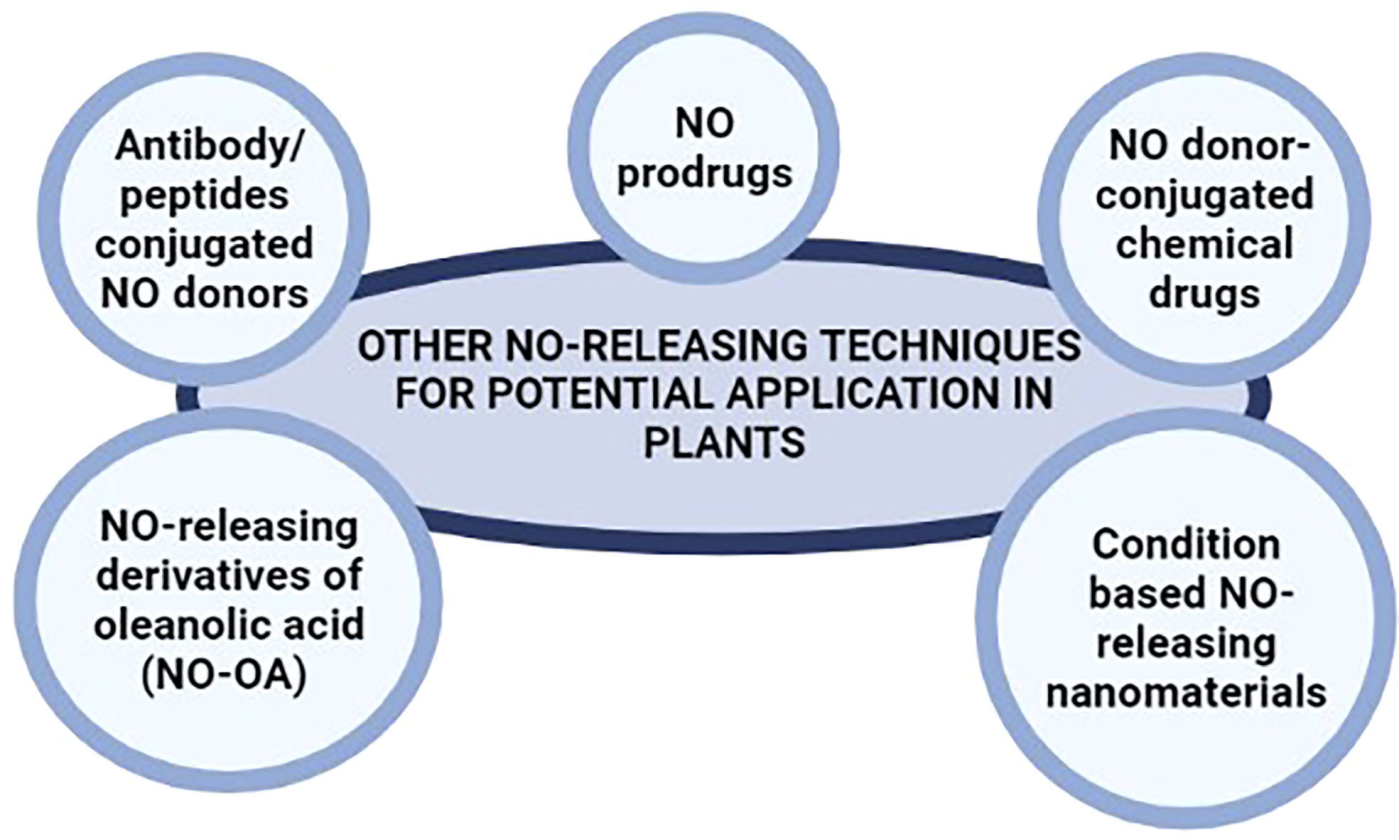
NO-releasing techniques used in biomedical research that may be potential alternatives to NO donors used in plant.

Similarly, drug delivery systems based on monoclonal antibodies also offer high target specificity in mammals. Antibody/peptides conjugated NO donors have been widely used in cancer treatment (Sievers and Senter, 2013; Chari et al., 2014; Sun et al., 2019) offering significantly higher specificity and release of NO following the detection of target cells only (such as cancer cells) by the monoclonal antibodies. NO donors conjugated to monoclonal antibodies can be engineered for the targeted, specific, and safe release of NO in plant systems under various circumstances. Such monoclonal antibodies can be tailored to recognize specific fungal, bacterial, and viral peptides (during infection), receptor proteins for various phytohormones (for regulating plant development and responses to various abiotic stresses), and several other peptides with spatial and temporal expression profiles; for the targeted delivery of NO in plant systems. **Figure 3** summarize the suggested NO-releasing techniques for potential application in plants.

## Conclusions and future prospects

Heavy metals occur naturally in the earth’s crust. They are often needed in very small amounts to carry out essential role in the metabolic systems of living organisms. However, natural calamities like weathering of rocks and volcanic eruptions, and other anthropogenic activities like mining and industrialization have largely overwhelmed their natural geochemical cycles (Nriagu and Pacyna, 1988). As a result, their concentration has increased in agricultural lands which negatively affects the growth and productivity of crops. To make things worse the application of chemical fertilizers containing heavy metals has further deteriorated the soil profile of agriculturally useful lands (Curtis and Smith, 2002).

Exposure of plant roots to heavy metals like cadmium (Cd), arsenic (As), lead (Pb), and copper (Cu), enhances endogenous levels of NO. NO is involved in various physiological and biochemical processes in plants that ensure optimal growth and development of plants exposed to various environmental stress conditions. Exogenous application of NO in the form of NO donors has been reported to lower oxidative stress by enhancing the activity of antioxidative enzymes under various environmental stress conditions. However, the use of NO donors is disadvantageous due to the short half-life of NO and its degradation by heat and light. A potential strategy is to use nanoparticles as encapsulating agents to effectively release NO in the plants for their optimal growth in heavy metal contaminated soils. Furthermore, to alleviate the toxicity caused by heavy metals in plants, NO needs to be delivered efficiently and for a prolonged duration. Therefore, conjugating them with the right nanoparticle is an important consideration. In this context, chitosan nanoparticles are suggested to be the most suitable candidates for this purpose owing to their unique properties such as biodegradable and biocompatible nature. Therefore, these nanoparticles need to be tested for their role in mitigating heavy metal stress in plants to sustain agricultural productivity. In conclusion, any NO-releasing technique that promises prolonged and efficient delivery of NO in an eco-friendly manner has the potential of alleviating heavy metal toxicity in plants.

## Author contributions

AP: conceptualization, visualization, writing - original draft. B-GM: project administration. NM: resources. WR: investigation. D-SL: visualization. GML resources. JH: writing - review and editing. AH: writing - review and editing. GL: final review and editing. B-WY: supervision, funding acquisition. All authors contributed to the article and approved the submitted version.

## Funding

This research was supported by Basic Science Research Program through the National Research Foundation of Korea (NRF) funded by the Ministry of Education (Grant number 2020R1I1A3073247), Republic of Korea and Korea Basic Science Institute (National Research Facilities and Equipment Center) grant funded by the Ministry of Education (NRF-2021R1A6C101A416).

## Acknowledgments

The graphical abstract and **Figures 1** and **3** were made in BioRender (BioRender.com).

## Conflict of interest

The authors declare that the research was conducted in the absence of any commercial or financial relationships that could be construed as a potential conflict of interest.

## Publisher’s note

All claims expressed in this article are solely those of the authors and do not necessarily represent those of their affiliated organizations, or those of the publisher, the editors and the reviewers. Any product that may be evaluated in this article, or claim that may be made by its manufacturer, is not guaranteed or endorsed by the publisher.
